# “The New State That We Are Building”: Authoritarianism and System-Justification in an Illiberal Democracy

**DOI:** 10.3389/fpsyg.2021.703280

**Published:** 2021-09-06

**Authors:** Jan-Erik Lönnqvist, Zsolt Péter Szabó, László Kelemen

**Affiliations:** ^1^Swedish School of Social Science, University of Helsinki, Helsinki, Finland; ^2^Department of Ergonomics and Psychology, Budapest University of Technology and Economics, Budapest, Hungary; ^3^Department of Social and Organizational Psychology, University of Pécs, Pécs, Hungary

**Keywords:** authoritarianism, system justification, system threat, immigration attitudes, partisanship

## Abstract

The authoritarian personality is characterized by unquestionining obedience and respect to authority. System justification theory (SJT) argues that people are motivated to defend, bolster, and justify aspects of existing social, economic, and political systems. Commitment to the *status quo* is also a key characteristic of the authoritarian personality. It can be argued that the social context matters for how an underlying latent authoritarian character is expressed. This means that authoritarian regimes could be expected to lead to increased authoritarianism and stronger system-justification. We investigated this hypothesis in two representative samples of Hungarians, collected before (2010) and after (2018) 8 years of Fidesz’ rule (*N* = 1,000 in both samples). Moreover, the strong version of SJT argues that members of disadvantaged groups are likely to experience the most cognitive dissonance and that the need to reduce this dissonance makes them the most supportive of the *status quo*. This argument dovetails nicely with claims made by the political opposition to Fidesz, according to which Fidesz is especially popular among low-status members of society. We found that measures assessing authoritarian tendencies did not change between 2010 and 2018. However, more specific beliefs and attitudes did change, and these effects were especially pronounced among Fidesz supporters. Their belief in a just world and a just system has grown stronger, while their attitudes toward migrants had hardened. Low status was associated with lower levels of system-justifying ideologies. However, low status Fidesz voters justified the system more than high status opposition voters in 2018, lending some support for the strong version of SJT. Our results suggest that beliefs and attitudes of Hungarians have changed between 2010 and 2018, and that political leadership played a crucial role in this.

## Introduction

“… The defining aspect of today’s world can be articulated as a race to figure out a way of organizing communities, a state that is most capable of making a nation competitive. This is why, honorable ladies and gentlemen, a trending topic in thinking is understanding systems that are not Western, not liberal, not liberal democracies, maybe not even democracies, and yet making nations successful” (Viktor Orbán, Hungary’s Prime Minister, speech at the XXV. Bálványos Free Summer University and Youth Camp, 26th July, 2014).

One of the most influential social science books of the 20th century was the Authoritarian Personality by Adorno et al. (1950). They attempted to explain fascism, the Second World War and the Holocaust with psychological factors as active forces in the social process. Their conceptualization of the authoritarian personality built on Fromm’s definition of the authoritarian character as a type of personality that loves authority – a personality type that simultaneously wishes to be in authority and to be subject to the will of the authority. They argued that this type of personality, which itself was partly a result of hierarchical and authoritarian parent-child relationships, made a person easily susceptible to anti-democratic propaganda and a potential fascist.

The current global rise of autocratization and retreat of democracy (Lührmann and Lindberg, 2019) has spurred research interest and led to new approaches and new measures of the authoritarian personality. The authors of the *Authoritarian Personality* argued that the social context matters for how an underlying latent authoritarian character is expressed in authoritarian attitudes and authoritarian behavior. More recent theoretical work has suggested an explanatory role for social learning (Altemeyer, 1981, 1988, 1996). However, very little is known about the role of institutions in the psychology of the individual. More specifically, very few empirical studies have investigated changes in authoritarianism in response to antidemocratic shifts in the political system. In the present study, we address this gap in the literature by investigating authoritarian characteristics, including right-wing authoritarianism (RWA; Duckitt and Sibley, 2007) and system-justifying beliefs (Kay and Jost, 2003), in two representative samples of Hungarians, collected before and after 8 years of an “illiberal democracy.”

An important rationale underlying the generation of the new illiberal state was the establishment of a national economic elite and protection of the middle class. However, the opposition to Fidesz has argued that Fidesz is especially popular among those low in social status (Csekõ, 2020). This makes it a particular interesting context in which to investigate whether those low in social status will engage in system justification, apparently against their own interests, but as suggested by the strong version of system justification theory, according, to which “people who are the most disadvantaged by a given social system should paradoxically be the most likely to provide ideological support for it, insofar as they have the greatest need to justify their suffering” (Jost et al., 2004, p. 267).

In the following introduction, we first sketch the major developments in the Hungarian political system over the past decade, then we introduce the two theoretical frameworks upon which we rely, namely, the authoritarian personality and system justification theory. Finally, we present our research questions.

### The Rise of the Illiberal Democracy: The Hungarian Context

Following the collapse of the Soviet Union in 1989, Hungary was a leader in the region’s liberal transition and among the first post-communist nations to join the European Union in 2004 (Lendvai, 2012). However, the transition to a marker-based economy was a far more protracted and difficult process than many observers initially expected. Hungary suffered badly in the 2008 financial crisis and was on the verge of defaulting until the International Monetary Fund, demanding stringent austerity measures, provided a bailout package. In 2006, a social democrat party speech was leaked, in which the then prime minister, Ferenc Gyurcány, admitted having lied repeatedly about the condition of Hungary’s public finances, and said that the economy could no longer sustain his party (MSZP)’s promises. This led to several weeks of mass demonstrations, calls for resignation, and violent clashes with the police (Lendvai, 2012).

The public spending crisis and a growing constituency of socioeconomically harassed voters allowed Fidesz, the largest opposition party, to run a notably blank electoral campaign in 2010 in terms of economic issues. Instead, they ran on a culturally conservative and nationalist platform. In their election program, the charismatic party leader Viktor Orbán pledged to increase police presence, raise prison sentences, assist families to have more children, protect marriage as the union between a man and a woman, protect life from the moment of conception, and honor the elderly (Batory, 2010).

In the 2010 elections, Fidesz won 53% of the popular vote. Due to the strong majoritarian element of the electoral system, this was enough to give it a 68% majority in parliament. The two-thirds majority of parliament allowed Fidesz to make major institutional changes. They employed this legislative dominance by changing the constitution and by replacing key officials in every politically relevant institution. Fidesz’s illiberalism was reflected in both the nature of the institutional reforms and the practices through which the party governed (Pogány, 2013). Public broadcasting and the national news agency were subsumed under the authority of a new government-dominated body. Fidesz also used its dominant legislative position to pave the way for gerrymandering and for making the electoral system even more majoritarian. Consequently, Fidesz secured a two-thirds parliamentary majority in both the 2014 and the 2018 national elections.

In terms of economic policy, Fidesz has advocated a “bourgeoisification” of the country with the aim of creating a middle- and upper-class who would regard Fidesz as their natural political party (Wilkin, 2018). A raft of policies sometimes referred to as “Orbanomics” included redistributive strategies to shield middle-class Hungarian voters from the pressures of unrestrained capitalism. It also contained numerous illiberal elements, such as increasing state ownership of enterprises in the banking, advertising, and transportation industries. This was not “state capture” carried out by a small group of oligarchs in order to establish regulations and pass measures in their own interests. Rather, the process ran in the other direction: Orbán decided who should become an oligarch and how powerful he should be (Kornai, 2015). András Lánczi, president of the Fidesz-leaning think-tank Századvég, famously said that “What (the critics of Orbán regime) call corruption in practical terms is the most important policy goal of Fidesz. What do I mean? The government puts forth such goals as the creation of a domestic entrepreneurial class, or the building of the pillars of a strong Hungary in agriculture and industry” (Antal, 2019). As a result of these economic measures (including the introduction of a flat tax and curbs to social benefits) and external factors (e.g., funding from the European Union, global economic revival; Kingsley and Novak, 2018), Hungary has, since 2012, enjoyed one of the highest rates of economic growth in the EU, accompanied by the highest increase in the risk of in-work poverty (Albert, 2019).

Despite the economic crisis in the run-up to the 2010 elections, the deeper causes of Fidesz success may have been more due to social than economic issues (Mudde, 2014). The ethnically exclusive and intolerant form of Hungarian national identity on which Fidesz has campaigned since 2010 (Marsovszky, 2010) has manifested itself in Orbán’s pronouncements on Hungary as part of a Central European migrant-free zone, which has successfully thwarted both cultural globalization and an influx of foreigners (Wilkin, 2018). The fight against “illegal immigration” has become a key element of Fidesz’s program since 2015. In a 2017 speech, Orbán said that “The truth is that now (…) everything that we think about Hungary and the order of life in Hungary is once again under threat. The truth is that after we regained our freedom in 1990, we have once again arrived at a crossroads in our history. (…) And now here we are, astonished to see that the forces of globalism are trying to force our doors open (…) We alone resist them now. We have reached the point at which Central Europe is the last migrant-free region in Europe.” His message could not be clearer: the traditional Hungarian way of life is in danger and must be defended.

The political changes of the last decade have resulted in a political system in which the degree of power concentration is exceptional. According to rating agencies, such as Freedom House, the Bertelsmann Foundation, the World Bank, and the Economist Intelligence Unit, Orbán has successfully hollowed out Hungary’s democracy. Since Fidesz won the elections in 2010, Hungary has become the clearest example of a relatively stable democracy turning into an authoritarian regime (Levitsky and Way, 2020; for similar observations, see Bogaards, 2018; Kelemen, 2019), and to have decayed from democracy into competitive authoritarianism, defined as “a type of regime in which the coexistence of meaningful democratic institutions and serious incumbent abuse yields electoral competition that is real but unfair” (Levitsky and Way, 2020, p. 51). Orbán openly stated his preference for an illiberal state in a July 2014 speech, in which he encouraged his Hungarian audience to understand systems that are “not Western, not liberal, not liberal democracies, maybe not even democracies, and yet making nations successful” (Rupnik, 2016).

In summary, since 2010, the Hungarian political and economic context has been characterized by: (1) strong economic growth; (2) economic measures that favor the middle- and the upper-class at the cost of the lower or working classes; (3) an authoritarian turn; i.e., a move from democracy into competitive authoritarianism; and (4) system threat induced by “illegal immigration.”

### The Authoritarian Personality

Some of the many attempts to explain the rise of fascism drew on the psychology of the individual. Most notable was Adorno et al.’s (1950) *The Authoritarian Personality*. Seeking to discover the psychological roots of social intolerance, the authors argued that the fascistic individual was psychologically susceptible to the ideology of anti-Semitism and to the emotional appeal of anti-democratic politics. They identified a personality syndrome that supported conventional values and authoritarian submission, as well as authoritarian aggression toward “inferior” minority groups, who were thought of judgmentally, harshly, and rigidly.

Although the psychoanalytic basis on which Adorno et al. (1950) constructed their theory has been highly criticized and tends to be ignored, the general tenet that right-wing political orientation can be correlated with certain underlying psychological dispositions has held up well and continues to attract attention. Subsequent research has confirmed that the social psychological and behavioral processes thought to constitute the authoritarian personality syndrome, conventionalism, conformity, cynicism, moral absolutism, intolerance and prejudice, tend to bundle together (e.g., Jost et al., 2003). One of the most prominent contemporary theories of authoritarianism was developed by Altemeyer (1981; (1988; (1996), who coined the term right-wing authoritarianism, to refer to aggression, submission, and conventionalism. The conceptual and methodological narrowing down of the original aspects of authoritarianism allowed Altemeyer to develop the RWA scale, which measured a strong unitary social attitude dimension, making it psychometrically superior to the original F-scale developed by Adorno et al. (1950). However, as Feldman (2003) argued the concept still lacked secure theoretical grounding and he went on to suggest a new conceptualization in which authoritarian predispositions originated in the conflict between the values of social conformity and personal autonomy. An overarching theme across conceptualizations is that authoritarianism underlies prejudice (e.g., Duckitt and Sibley, 2007).

Although authoritarianism is a complex attribute, most definitions of the concept seem to agree that it comprises simultaneous dominance of inferiors and submissiveness to superiors. It is remarkable how well such a core definition dovetails with the political program of Fidesz. On one hand, Fidesz stirs up hatred toward disadvantaged minorities, such as refugees (Krekó and Enyedi, 2018), Muslims (Kende et al., 2019), and sexual minorities (Bene and Boda, 2021). On the other hand, Fidesz propagates submission to those in power, emphasizing, e.g., traditional gender roles, which place women in inferior positions to men (Vida, 2019). Most importantly, they require total political submission to the “homeland” and its “people,” crushing subnational capacity for institutional resilience, destroying the independent judiciary, and taking full control of the media landscape (Jakli and Stenberg, 2021; Seongcheol, 2021).

### The Role of Institutions in Authoritarianism

The rise of the European populist radical right and the 2016 United States election of Donald Trump has resulted in a resurgence of research into authoritarianism (Nilsson and Jost, 2020). Much of this research has, however, been confined to predicting political orientation and voting behavior from authoritarian personality characteristics, with far less attention given to the causes of authoritarianism (e.g., Dunn, 2015). One approach to “explaining” authoritarianism has been to show that individual differences in authoritarianism are genetically or biologically based (e.g., McCourt et al., 1999). Few would probably dispute this altogether, as the development of most characteristics is likely to involve genes and the interaction between genes and environment. Yet, asserting that authoritarianism is simply inborn does little to explain large cross-national and across time differences in authoritarianism. Another prominent approach has been to explain authoritarianism as the product of social learning, or more specifically, the result of one’s individual experiences with authority (e.g., Altemeyer, 1981, 1988, 1996). These perspectives are of course not mutually exclusive. As already argued by Frenkel-Brunswik et al. (1947), authoritarian characteristics portray a latent capacity or “degree of readiness to behave antidemocratically should social conditions change in such a way as to remove or reduce the restraint upon this kind of behavior” (Frenkel-Brunswik et al., 1947, p. 40).

Consistent with the notion that changing social conditions may unleash antidemocratic behaviors, some recent electoral outcomes have been claimed to have loosened moral and ethical restraints and normalized violent lawlessness. It has been argued that the election of Trump returned “the scourge of authoritarianism (…) not only in the toxic language of hate, humiliation and bigotry, but also in the emergence of a culture of war and violence that looms over society like a plague” (Giroux, 2017, p. 887). There is, indeed, some empirical evidence suggesting that Trump’s popularity on the campaign trail and subsequent election win increased people’s willingness to publicly express xenophobic views (Bursztyn et al., 2017), and the acceptability of prejudice toward groups Trump targeted (e.g., Crandall et al., 2018; Hobbs and Lajevardi, 2019). Anti-Muslim crimes have doubled since Trump’s presidential campaign, with some analysis suggesting that Trump’s tweets about Islam-related topics (Müller and Schwarz, 2020) were directly responsible for certain crimes. One of the main aims of our study was to investigate whether, in a similar vein, Hungary’s authoritarian descent has been accompanied by an authoritarian slide in the populace.

Besides studies investigating how the outcomes of specific elections may unleash authoritarian behaviors, there have been some cross-cultural studies that have sought to determine the influences that different types of regimes may have on the individual’s psychology. Some of these studies have suggested that commitment to democratic principles and rejection of authoritarian alternatives is higher in democratic than in authoritarian political regimes (Chu et al., 2008; Mujani and Liddle, 2013), although such studies (and others) also expose substantial variations within both democratic and authoritarian regimes (e.g., Inglehart et al., 2003; Chang et al., 2013; Shin, 2015; Park, 2017), thereby leaving open the question of whether there is a consistent impact of regime on particular citizens’ political value orientations.

One reasons for these inconsistent results regarding political preferences in different political systems could be that this research has to large extent relied on country level-comparisons. Unfortunately, these comparisons tend to be tainted by measurement problems. For instance, language systematically affects the meaning and interpretation of survey items, and different responses will be given depending on the language of the item (Pérez, 2011). There are also other country-biases than language (e.g., popular conceptions of the meaning of the word “democracy” vary between countries; Chu and Huang, 2010), meaning that comparisons between countries will generally be grossly misleading.

Some studies that have looked at socialization within a given culture suggested that citizens socialized under authoritarian rule are less supportive of democracy than those socialized under democratic rule (Pop-Eleches and Tucker, 2014; Voicu and Bartolome Peral, 2014). One study, focusing on the individual, found that preferences for democracy increase as individuals experience more time living under democratic rule (Fuchs-Schündeln and Schündeln, 2015), although single culture studies have given mixed results (e.g., Haerpfer and Kizilova, 2014). Besides being scant, an important limitation of the present literature connecting changes in political regimes with the political preferences of the individual is that it very much focuses on the shift from authoritarian forms of government toward democracy. There is very little research on what happens when the direction of change is the opposite; that is, from democracy toward authoritarianism.

### System Justification in an Authoritarian State

The central tenet of system justification theory is that people have a basic motivation to legitimize the social system (Jost and Hunyady, 2005). System justification meets epistemic, existential, and relational needs, providing individuals with a sense of security and enables them to maintain a shared reality with others while alleviating their sense of external threat.

Authoritarianism and system justification are closely associated constructs (Wilson and Sibley, 2013; Osborne and Sibley, 2014) – they share an attachment to “things as they are,” a resistance to social change, and an ideological commitment to the *status quo*, religion, and tradition (see also Jost and Kende, 2020). Jost and Hunyady (2005) used the umbrella term system-justifying ideologies to describe a set of worldviews (e.g., just world beliefs, Protestant work ethic, meritocratic ideology, fair market ideology) of which one specific type is right-wing authoritarianism. The authors argued that these ideologies serve to legitimize the prevailing social order. Importantly, however, previous empirical studies have found only zero or very low correlations between individual difference measures of RWA and of system justification. For example, Osborne and Sibley (2014) observed a low positive correlation in an American sample, while Kelemen et al. (2014) obtained a low negative correlation in a Hungarian sample surveyed during a period of left-wing government in Hungary (for a similar result in France, see Langer et al., 2020). In a cross-cultural study, Vargas-Salfate et al. (2018) found a low within-country individual level positive association between RWA and system justification (*r* = 0.169), but nothing at the country level (*r* = 0.106).

Besides direct measures of authoritarianism and system justification, we also measured belief in a just world (BJW; Lerner and Miller, 1978) and immigration attitudes. We now explain our reasons for doing so. BJW refers to the belief that everyone gets what they deserve (Lerner and Miller, 1978), and can be considered a system justifying ideology. Those who believe the world to be just will perceive the *status quo* as legitimate and believe that there is no need for social action or for social change (Hafer and Choma, 2009).

Regarding immigrant attitudes, immigrants have, by Fidesz and the Fidesz dominated state media, been consistently portrayed as a danger to Europe and the greatest threat to the Hungarian nation (Bocskor, 2018). A sense of perceived threat is at the very core of both the authoritarian personality and of system justifying ideology. Indeed, increased authoritarianism and system-justification go hand in hand with increasing levels of perceived symbolic or material threats to the *status quo* (Kay and Zanna, 2009; Kay and Friesen, 2011). In the Hungarian context, in which migrants have been portrayed as the greatest threat, increases in authoritarianism and system justification would be expected to tally with hardened attitudes toward migrants. Moreover, the perceived threat imposed by migrants could, as explained below, help explain why low social status Fidesz voters may support a system that has made them worse off.

### The Strong Version of System Justification

One key strength of system justification theory is that it plausibly explains why people show support for social systems that oppose their personal or group-based interests. The strong or dissonancy-based version of system justification theory (also referred to as the status-legitimacy hypothesis; see Brandt, 2013, p. 2) can also explain why members of disadvantaged groups legitimize the *status quo*, and why they do not engage in system-challenging collective action (e.g., Osborne et al., 2019; De Cristofaro et al., 2021). Owuamalam et al. (2019), although acknowledging that system justification theory is more than the system justification motive, argue that providing empirical evidence for the dissonance-based strong version serves as the “litmus test” of system justification theory. Such evidence is needed to support an independent motive for system justification and distinguished the theory from interest-based theories such as social identity theory (SIT) or self-categorization theory (SCT). Indeed, as Owuamalam et al. (2019) noted, several studies to test the strong version have been run, including studies striving to explain such paradoxical phenomena as working-class conservatism, low-income groups’ relatively strong preference for meritocratic ideologies, and their idealization of capitalism (Jost et al., 2003). The findings obtained in these studies are mixed in terms of the support they afford the strong version of the theory. Jost et al. (2003) showed that several low-status groups engage in system justification against their own personal and group-based interests, as reflected in their endorsement of income inequalities and meritocratic ideologies. Henry and Saul (2006) found evidence for the strong version of the theory in Bolivia, which is one of the poorest countries in the world. They found that children from low-SES Bolivian families strongly believed in the effectiveness of the government in meeting the people’s needs. These results are consistent with Jost et al. (2004) notion that the people who are the most disadvantaged by a given social system have the greatest need to decrease dissonance by justifying their suffering, and should, paradoxically, thus be the most likely to ideologically support the system (Henry and Saul, 2006, p. 267). Although there are many findings that do not support the strong version (e.g., Caricati and Lorenzi-Cioldi, 2012; Brandt, 2013; Kelemen et al., 2014; Caricati, 2017; Vargas-Salfate et al., 2018), Jost (2019) points out that also null results beg the question of why the lower classes are as likely or almost as likely to opt for the *status quo* as are the higher classes?

### The Present Research

In the 2010 parliamentary elections, Hungary witnessed political upheaval. MSZP, the ruling socialist party plummeted from 48% of the vote in 2006 to 15%, handing over victory to the two opposition parties, Fidesz and Jobbik. In the 2 months leading up to the elections, we assessed authoritarianism and system-justification in a nationally representative sample of 1,000 Hungarian adults (Lönnqvist et al., 2019b).

In 2018, we sought to rerun the same survey with new participants after 8 years of Fidesz rule. More specifically, using the same methodology as in 2010, we again surveyed a representative sample of 1,000 Hungarians. This was done 2 months after the 2018 elections. The state apparatus and the governing party had campaigned in tandem to give Fidesz 49% of the vote with an impressive 70% turn-out, thereby setting up its third straight two-thirds majority. Jobbik held onto its base with 19% of the vote. The divided leftist and liberal parties were unable to increase their share of votes. The central question of the current study was whether the authoritarian turn had been accompanied by changes in attitudes and beliefs as measured at the individual level. Authoritarianism of the populace could be expected to increase under 8 years of an illiberal democracy. This could be expected to be especially true among those who support the illiberal democracy. Our first hypothesis is therefore that this political turn would have strengthened authoritarianism (H1a), and especially among the voters of the ruling party (H1b).

As right-wing authoritarianism shares common features with system justification and just world beliefs, we also expected that system justification and personal and global just world beliefs would have also increased in the past 8 years (H2a), and especially among the voters of the ruling party (H2b).

The masses tends to adjust their attitudes to leadership cues (Zaller, 1992; Gabel and Scheve, 2007), and especially those who identify with a party tend to modify their issue stances to conform to their party (Carsey and Layman, 2006; Dancey and Goren, 2010; see also Lönnqvist et al., 2019a). This means that that Fidesz’s ever-increasing hostility toward immigrants, in part fueled by the so called “refugee crisis” in 2015, could have moved the populace’s attitudes in a more anti-immigrant direction. Our third hypothesis is that Fidesz’s hostility toward immigrants would have hardened attitudes toward migrants (H3a), and especially among the voters of the ruling party (H3b).

This context, in which intense economic progress is associated with aggravated inequalities and an increased risk of marginalization. provided a unique context in which to test the strong version of system justification theory. Would disadvantaged group members justify a new social system that perpetuated their disadvantages? Did even low SES Fidesz supporters believe in the system? (RQ1).

## Materials and Methods

### Participants and Procedure

The 2018 study was designed as an exact replication of the 2010 study. Both were run with nationally representative sample of 1,000 Hungarian adults. The quotas (i.e., age, sex, education, and place of residence), were based on the most recent Hungarian Statistical Office data and the data was collected applying the random walking method. Overall, 3,980 Hungarian adults were approached by trained market researchers in 2010 and 4,095 in 2018. One thousand face-to-face interviews were successfully conducted both in 2010 (25%) and 2018 (24%). 1,229 people refused to participate in 2010 (31%) and 1,427 in 2018 (35%). 1,751 people did not conform to the quotas employed in 2010 (44%) and 1,668 in 2018 (41%). The participants, who did not receive any material compensation, were informed that the data collection was voluntary and anonymous. The 2010 and 2018 final samples both consisted of 1,000 Hungarian adults (527 females in 2010 and 526 females in 2018) with a mean age of 45.4 (SD = 16.5) in 2010 and 45.7 (SD = 16.9) in 2018. Regarding highest attained education, 47 participants had not finished elementary school in the 2010 sample and 23 in the 2018 sample, 436 had finished elementary school in the 2010 sample and 435 in the 2018 sample, 380 had finished high school in the 2010 sample and 435 in the 2018 sample, and 137 had finished higher education (BA or MA) in the 2010 sample and 134 in the 2018 sample. Two participants in 2018 did not answer this question. Data were collected in early 2010, some months before the April elections in which Fidesz came to power, and late in 2018, around half a year after Fidesz had won its third consecutive super-majority in parliament. In general, the two samples look essentially identical in terms of response rate, sex, age, and education. We conducted the research with the IRB approval of Eötvös Loránd University.

### Measures

All scales in the studies were abridged Hungarian adaptations (see Kelemen et al., 2014), and all items were responded to on a scale ranging from 1 (absolutely disagree) to 4 (absolutely agree).

#### Authoritarian Personality

Characteristics reflecting authoritarian personality were measured with scales assessing Authoritarianism [two items from the original F-scale (Adorno et al., 1950)] and two items added by Kelemen et al. (2014). The two Authoritarianism items from the original F-scale by Adorno et al. (1950) were (1) People can be divided into two distinct classes: the weak and the strong, (2) Human nature being what it is, there will always be war and conflict. The two items added by Kelemen et al. (2014) were (3) Everybody has to know his or her place in life in terms of both superiority and inferiority, and (4) It is both important to know how to obey and how to command. Cronbach’s alpha internal consistency reliabilities were 0.57 and 0.62 in 2010 and 2018, respectively.

#### Just World Beliefs

The abridged version of Lerner and Miller (1978) scale was used to measure global just world beliefs (GBJW) and personal just world beliefs (PBJW). The three GBJW items were: (1) I think basically the world is a just place, (2) I believe that, by and large, people get what they deserve, and (3) I am confident that justice always prevails over injustice. Alphas were 0.67 and 0.74 in 2010 and in 2018, respectively.

The four PBJW items were: (1) I think that important decisions that are made concerning me are usually just, (2) I believe that I usually get what I deserve, (3) In my life injustice is the exception rather than the rule, and (4) I believe that most of the things that happen in my life are fair. Alphas were 0.80 and 0.85 in 2010 and in 2018, respectively.

#### System Justification Beliefs

System justification was measured with Kay and Jost (2003) system justifying belief (SJB) measure. The five SJB items were (1) In general, I find society to be fair, (2) Hungarian society needs to be radically restructured (R), (3) Most policies serve the greater good, (4) Everyone has a fair shot at wealth and happiness, and (5) Our society is getting worse every year (R). Alphas were 0.67 and 0.78 in 2010 and in 2018, respectively.

#### Anti-immigration Attitudes

The six items assessing anti-immigration attitudes were: (1) We should defend our way of life from outside (foreign) influence, (2) Life is enriched by lots of different people living next to each other (R), (3) We should be stricter regarding the rights of people who want to live here, (4) It is good that the countries of the world are increasingly more connected (R), (5) The presence of foreigners increases the crime rate, and (6) Greater freedom in movement and settlement is beneficial for everyone (R). Alphas were 0.80 and 0.78 in 2010 and in 2018, respectively.

#### Party Affiliation

Regarding party affiliation, participants were asked which party they would vote for in case there were elections “next Sunday.” We formed five groups according to voting intentions (see Table 1). In 2010, the group socialists consisted of the MSZP (Hungarian Socialist Party), whereas it in 2018 consisted of 46 MSZP and 43 Democratic Coalition (DK) voters. The latter was formed in 2010 as a fraction of the MSZP by the then leader of the MSZP, current leader of the DK, under whose leadership it split away in 2011 to form a separate party, taking many of the MSZP voters with him. Voters of small political parties (*N* < 50) were not considered.

**TABLE 1:** Descriptive statistics and effect sizes for comparison between 2010 and 2018 means according to voting intention.

	Authoritarianism	Global just world beliefs	Personal just world beliefs	Anti-immigrant attitudes
Fidesz supporters				
2010 M (SD) *n* = 334	3.08 (0.48)	2.33 (0.61)	2.57 (0.60)	2.69 (0.58)
2018 M (SD) *n* = 327	3.08 (0.49)	2.69 (0.65)	2.83 (0.57)	3.13 (0.51)
SE	0.04	0.05	0.05	0.04
*d* (*p*)	0 (0.86)	−0.571 (<0.001)	−0.444 (<0.001)	−0.805 (<0.001)
95% CI	−0.84, 0.70	−0.46, −0.26	−0.35, −0.17	−0.53, −0.36
**Jobbik supporters**				
2010 M (SD) *n* = 124	3.19 (0.51)	2.13 (0.68)	2.56 (0.63)	2.92 (0.58)
2018 M (SD) *n* = 80	2.93 (0.49)	2.68 (0.78)	2.76 (0.63)	3.05 (0.62)
SE	0.07	0.10	0.09	0.08
*d* (*p*)	0.519 (<0.001)	−0.752 (<0.001)	−0.317 (0.021)	−0.217 (0.086)
95% CI	0.12, 0.40	−0.74, −0.36	−0.37, −0.03	−0.30, 0.02
**Social democrat supporters**				
2010 M (SD) *n* = 95	3.11 (0.52)	2.35 (0.69)	2.53 (0.57)	2.63 (0.63)
2018 M (SD) *n* = 89	3.12 (0.42)	2.55 (0.71)	2.64 (0.63)	2.82 (0.56)
SE	0.07	0.10	0.09	0.08
*d* (*p*)	−0.021 (0.89)	−0.286 (0.05)	−0.183 (0.207)	−0.319 (0.022)
95% CI	−0.16, 0.14	−0.39, 0.00	−0.29, 0.06	−0.35, −0.03
**Undecided**				
2010 M (SD) *n* = 177	3.11 (0.49)	2.31 (0.70)	2.55 (0.59)	2.64 (0.61)
2018 M (SD) *n* = 218	3.02 (0.55)	2.49 (0.69)	2.61 (0.62)	2.76 (0.55)
SE	0.05	0.07	0.06	0.06
*d* (*p*)	0.172 (0.08)	−0.259 (0.007)	−0.099 (0.343)	−0.189 (0.039)
95% CI	−0.01, −0.19	−0.32, −0.05	−0.18, 0.06	−0.23, −0.01
**No response**				
2010 M (SD) *n* = 206	3.06 (0.51)	2.34 (0.64)	2.50 (0.56)	2.70 (0.58)
2018 M (SD) *n* = 209	3.03 (0.52)	2.50 (0.74)	2.68 (0.63)	2.80 (0.50)
SE	0.05	0.07	0.06	0.06
*d* (*p*)	0.058 (0.513)	−0.231 (0.015)	−0.302 (0.002)	−0.185 (0.053)
95% CI	−0.06, −0.13	−0.29, −0.03	−0.29, −0.06	−0.22, 0.00

*Dependent variables: Authoritarianism, global just world beliefs, personal just world beliefs, anti-immigrant attitudes.*

#### Subjective Socioeconomic Status

Subjective socioeconomic status was measured with one question: “Which of the following statement characterizes your financial status the best?” The four options were: “I do not have any financial problems,” “I do not have financial problems, but I have to live within my means,” “I can’t buy everything I want, and usually run out of money before the end of the month,” and “I have serious financial troubles.” SES was collapsed to form two categories by combining the “I have serious financial problems” (*N* = 103 in 2010 and 31 in 2018) and the “I can’t buy everything I want, and usually run out of money before the end of month” (*N* = 421 in 2010 and 262 in 2018) categories to equal “low SES” with the other two options (“I do not have any financial problems,” *N* = 19 in 2010 and 52 in 2018; “I do not have financial problems, but I have to live within my means,” *N* = 457 in 2010 and 652 in 2018) classified as “high SES.”

### Data Analysis

To address our hypotheses (H1–H3), two-way ANOVAs were conducted that examined the effect of measurement time (2010 vs. 2018) and voting intentions (Fidesz vs. Jobbik vs. Social democrats vs. Undecided vs. No response) on the outcome variables (authoritarianism, global just world beliefs, personal just world beliefs, and anti-immigrant attitudes). To test our research question (RQ1), a three-way ANOVA was conducted that examined the effect of measurement time, voting intentions, and socioeconomic status on system justification beliefs. For pairwise comparisons, we used Bonferroni correction *post hoc* tests. Cohen’s *d* was calculated using the pooled standard deviation across groups.

## Results

A two-way ANOVA with authoritarianism as dependent variable, revealed a statistically significant interaction between the effects of measurement time and voting intentions on authoritarianism, *F*(4,1849) = 3.01, *p* = 0.017, η*_*p*_*^2^ = 0.006. Simple main effects analysis showed that Jobbik voters were significantly more authoritarian in 2010 than in 2018, but the authoritarianism of other voters did not change between 2010 and 2018 (*p*s > 0.05). In fact, Jobbik supporters had plummeted from being highest in authoritarianism in 2010 to being lowest in 2018. Table 1 reports the standard error associated with the estimated marginal means, the relative *p*s, the confidence intervals, and Cohen’s *d*.

A similar two-way ANOVA with global just world beliefs as dependent variable, revealed a statistically significant interaction between the effects of measurement time and voting intentions on global just world beliefs, *F*(4,1849) = 1.813, *p* = 0.003, η*_*p*_*^2^ = 0.009. Simple main effects analysis showed that all groups in 2018 thought the world generally was more just than in 2010, and this effect was the strongest among Fidesz and Jobbik voters. Table 1 reports the standard error associated with the estimated marginal means, the relative *p*s, the confidence intervals, and Cohen’s *d*.

A two-way ANOVA with personal just world beliefs as dependent variable revealed a statistically non-significant interaction between the effects of measurement time and voting intentions on personal just world beliefs, *p* = 0.102. The main effects of measurement time, *F*(1,1849) = 26.624, *p* < 0.001, η*_*p*_*^2^ = 0.014, and voting intentions, *F*(4,1849) = 3.628, *p* = 0.006, η*_*p*_*^2^ = 0.008, were significant. Personal just world beliefs were lower in 2010 compared to 2018, and Fidesz voters had higher scores than the no response group and the undecided group. Table 1 reports the standard error associated with the estimated marginal means, the relative *p*s, the confidence intervals, and Cohen’s *d*.

A two-way ANOVA with anti-immigrant attitudes as dependent variable revealed a statistically significant interaction between the effects of measurement time and voting intentions on anti-immigrant attitudes, *F*(4,1849) = 15.623, *p* < 0.001, η*_*p*_*^2^ = 0.018. Simple main effects analysis showed that all groups became more anti-immigrant, except Jobbik voters, who in 2010 were already very anti-immigrant, much more so than any other group. However, now Fidesz voters, showing a large increase in anti-immigrant attitudes, were as anti-immigrant as Jobbik voters. Other groups showed only small increases in anti-immigrant attitudes. Table 1 reports the standard error associated with the estimated marginal means, the relative *p*s, the confidence intervals, and Cohen’s *d*.

A three-way ANOVA with system-justification beliefs as dependent variable revealed a statistically significant three-way interaction between measurement time, voting intentions and socioeconomic status, *F*(4,1836) = 2.770, *p* = 0.26, η*_*p*_*^2^ = 0.006. Simple main effects analysis showed that both low SES and high SES Fidesz voters judge the system as more just in 2018 than in 2010. A similar pattern emerged for the No response (both low and high SES), the Undecided groups (both low and high SES), and the low SES Social democrat voters. High SES Jobbik voters also judged the system as more just in 2018 than in 2010. This was not the case for low SES Jobbik voters and high SES Social democrat voters. Largest differences were found among Fidesz voters. The main effect of SES was significant, *F*(1,1836) = 48.987, *p* < 0.001, η*_*p*_*^2^ = 0.026. Higher SES was generally associated with higher system justification scores both in 2010 and 2018. However, low SES Fidesz voters had higher SJB scores in 2018 than high SES other voters in 2018. Table 2 reports the standard error associated with the estimated marginal means, the relative *p*s, the confidence intervals, and Cohen’s *d*.

**TABLE 2:** Descriptive statistics and effect sizes for comparison between 2010 and 2018 means according to voting intention and SES.

	SES	2010 M (SD)	2018 M (SD)	SE	P	95% CI	Cohen’s *d*
Fidesz	Low	1.75 (0.46)	2.44 (0.56)	0.07	<0.001	−0.82, −0.56	1.346
	High	1.91 (0.46)	2.71 (0.53)	0.06	<0.001	−0.90, −0.69	1.612
Jobbik	Low	1.62 (0.44)	1.71 (0.43)	0.14	0.528	−0.38, 0.19	0.207
	High	1.71 (0.50)	2.19 (0.49)	0.10	<0.001	−0.68, −0.29	0.970
Social democrats	Low	1.82 (0.55)	2.06 (0.54)	0.14	0.089	−0.51, 0.04	0.440
	High	2.12 (0.55)	2.05 (0.55)	0.10	0.481	−0.13, 0.27	0.127
No response	Low	1.79 (0.55)	2.12 (0.59)	0.09	<0.001	−0.50, −0.15	0.579
	High	2.03 (0.48)	2.22 (0.63)	0.07	0.005	−0.33, −0.06	0.339
Undecided	Low	1.76 (0.44)	1.96 (0.64)	0.09	0.022	−0.36, −0.03	0.364
	High	1.97 (0.52)	2.23 (0.61)	0.07	<0.001	−0.41, −0.12	0.459

*Dependent variable: System justifying beliefs.*

*SES, Subjective socioeconomic status.*

## Discussion

We investigated the extent to which the Hungarian public had changed in terms of characteristics associated with authoritarianism and system justification after 8 years of Fidesz’s rule. Mean scores on our measures of authoritarianism were generally stable over time. These results do not support H1a and H1b.

The results were different for system-justifying belief and belief in a just world. Fidesz supporters, in particular, believed the world to be more just in 2018 compared with 2010, but also others believed the world to be more just. Fidesz supporters also stood out in terms of starkly increased belief in the system. However, everyone else, except supporters of the social democrats and low SES Jobbik voters, also thought the system was more just in 2018 than in 2010. These results support both H2a and H2b.

Regarding anti-immigrant attitudes in 2018, Fidesz supporters were much more anti-immigrant than in 2010, with other groups showing much smaller increases in anti-immigrant sentiment, supporting H3a and H3b.

Our results do not generally support the strong version of system justification theory: system justification was positively associated with socioeconomic status both in 2010 and 2018. However, in 2018, low status Fidesz voters were more prone to justify the system than were voters of other parties, including even high status voters of other parties. This lends some support for the strong version of SJT (RQ1).

### Stable Values and Changing Attitudes

In terms of contemporary personality research, our measures of authoritarianism could be argued to resemble personal values. Personal values are general conceptions of what is desirable; they are more abstract than attitudes since they transcend specific actions and situations (e.g., Schwartz, 1992). Our measures of authoritarianism could be argued to tap into valuing hierarchy over egalitarianism and submission over criticism, respectively. Although values are often thought to be malleable to culture and life events, they have, in fact, been shown to be remarkably stable in adulthood. For instance, vocational training or education in a certain discipline does little to influence such fundamental values (Bardi et al., 2014). Two longitudinal studies to have investigated value change in migration have suggested that values do change in response to such major life transitions (Lönnqvist et al., 2011; Bardi et al., 2014). However, it seems that these changes may be temporary; one of the studies included a 2-year follow-up, at which stage values had reverted back to their initial pre-migration levels (Lönnqvist et al., 2013).

Regarding the more direct question of whether the political system in a country influences personal value priorities, the empirical evidence is mixed. For more than four decades, the populace of Eastern Europe was subject to communist regime. However, these regimes and their symbols remained alien to the populace and were not generally accepted (Rupnik, 1988). For the period preceding communist rule, data on value priorities is scant. One study conducted in the early 1990s, after the fall of the communist regimes suggested that Eastern Europeans did not differ as a group from their Western counterparts in most values related to politics, religion, and primary relations (van den Broek and de Moor, 1994). By contrast, another study based on data from the same time period suggests that the communist system did move personal values toward higher hierarchy and conservatism and lower autonomy (Schwartz et al., 2000). Such cross-cultural comparisons are, however, hampered by lack of scalar invariance across countries (Davidov, 2010). This means that comparisons of mean importance across countries are likely to be highly misleading. Our results are generally consistent with the above literature; values, at least in the short run, do not seem to change in response to the political system.

Our results revealed increased anti-immigrant attitudes and system-justifying beliefs. This is consistent with a large body of research suggesting that the populace tends to adjust its attitudes to leadership cues (Zaller, 1992; Gabel and Scheve, 2007). Also consistent with previous research is that both beliefs and attitudes changed the most among Fidesz supporters. Since the 1960s, an impressive amount of literature on “the role of enduring partisan commitments in shaping attitudes toward political objects” (Campbell et al., 1960, p. 135; for a review, see Bartels, 2002) has accumulated. Those who identify with a party tend to modify their issue stances to conform to their party (Carsey and Layman, 2006; Dancey and Goren, 2010; see also Lönnqvist et al., 2019a). Other groups of voters showed only small increases in anti-immigrant sentiment. Nevertheless, although attitudes have in the last decades generally become more liberal, our results suggest that there is nothing inevitable about this (cf., Strimling et al., 2019). The process can be reversed, and political leadership may play a crucial role in such a reversal.

### Worldviews Changed, but Why?

There was, overall, a moderate increase in both global and personal belief in a just world, and a large increase in belief a just system. Belief in a just world and belief in a just system have been argued to be caused by factors such as insecurity (Jost et al., 2008), threats to the system and consequent instability (Jost and Hunyady, 2005), perceptions of a dangerous world (Jost and Hunyady, 2005), and needs for order, structure, closure, and control (Jost et al., 2003, 2017; Jost and Hunyady, 2005). It is conceivable that the fear mongering Fidesz’s leadership and of the state dominated media led people to experience heightened insecurity and threat, and that that heightened belief in the system and in a just world served a palliative function. Indeed, there is ample evidence that system justifying beliefs are associated with lower levels of anxiety, discomfort, and uncertainty (see Jost and Hunyady, 2003). Recently, Vargas-Salfate et al. (2018) showed, in a longitudinal 18-country study, that endorsing system-justifying beliefs is positively related to general psychological well-being.

On the other hand, other explanations than those referring to the palliative function of system justifying beliefs are possible. There was real moral outrage at the societal *status quo* in the run-up to the 2010 elections. After all, the then prime minister had been caught on tape admitting to having lied for years about the economy and Hungary’s economy had collapsed (Lendvai, 2012). Moral outrage at the *status quo* has been negatively associated with belief in just system (e.g., Wakslak et al., 2007; Becker and Wright, 2011; Jost et al., 2012), and moral conviction can overpower system-justifying beliefs (De Cristofaro et al., 2021). It is feasible that moral outrage targeted at the previous government served to strengthen system justification after Fidesz was voted into power. People’s belief in a just world and a just system may have hit a low-point in 2010, and the mere return to a more-or-less stable and normal way of life, allowed by strongly increasing levels of income, may have been enough to raise belief in a just world and a just system. Future research could try to disentangle the possible effects of state fear mongering from the effects of increased wealth as underlying increased just world and just system beliefs.

Belief in a just world, and even more so system-justifying belief, showed strong party-bias. It was primarily Fidesz supporters whose belief in a just world and a just system grew stronger. This result is consistent with a large body of research showing that partisan bias shapes not only more value-laden judgments (Bartels, 2002; Carsey and Layman, 2006; Dancey and Goren, 2010), but also more factual beliefs about the world; e.g., economic conditions (both current and future) are described as being better when the supported political party is in office (e.g., Gerber and Huber, 2010; Benhabib and Spiegel, 2019) or has just won an election (Gillitzer and Prasad, 2018). Given that even such, in some sense “objective” and factual beliefs are biased by partisanship, our results according to which more abstract beliefs regarding the world and the system are similarly biased cannot be considered surprising.

### Conflicting Evidence for the Strong Version of System Justification Theory

Our results do not generally support the strong version of system justification theory: system justification was positively associated with socioeconomic status. However, some findings deserve particular attention in terms of the strong version of system justification theory. Voters for the ruling party Fidesz included individuals who reported low SES (i.e., serious financial difficulties), and these low SES Fidesz voters showed a higher mean level of system justification than did opposition voters with high SES. That individuals struggling with serious or mild financial difficulties in a period of intense economic progress still support the system would seem to support the notion that “some of the ideas we hold are quite simply not good for us, and in that sense, they do not serve our interests or the interests of our ingroups”’ (Jost et al., 2019, p. 384). In this sense, our results do support the notion that disadvantaged-group members legitimize the *status quo* under certain conditions.

One explanation for even low SES Fidesz voters belief in the system could be the perception of immigrants as threatening. Our results show that anti-immigrant attitudes were positively correlated with system justification. Kay and Zanna (2009) argued that increasing terror alert in the United States after 09/11 could be viewed as a natural manipulation of system threat, and something similar could have happened in Europe with the so called “refugee crisis” of 2015. Supporting the idea that it was this external threat that could have contributed to low SES Fidesz’ voters endorsement of the system, both low and high SES Fidesz voters were harsher in their attitudes toward migrants than were other voters. The threat that immigrants imposed could be perceived as a controllable threat (by contrast to for instance the 2008 financial crisis, after which system derogation was commonplace in Hungary; see Kelemen et al., 2014; Szabó and Lönnqvist, 2021), and this perception may have been further supported by the continuous decrease in the number of those illegally entering Hungary over recent years, a pattern that Fidesz claimed credit for Bíró-Nagy (2021). Problematically, increased system justification among the disadvantaged can undermine system-changing collective action intentions (Osborne et al., 2019; De Cristofaro et al., 2021).

### Limitations and Conclusion

An obvious limitation of the present research is that the design was cross-sectional. A longitudinal design would have allowed us to assess to what extent between-party changes in attitudes were due to people changing their beliefs and attitudes or people changing party. For instance, we cannot tell whether 2010 Fidesz supporters became more anti-immigrant during the following years, or whether more anti-immigrant people became Fidesz supporters. However, Hungary has, after the volatility in the years leading up to the 2010 election, been characterized by stability of party politics (Enyedi, 2016). We thus believe that the changes that we observed, particularly in Fidesz supporters, were due to those supporters changing, and not due to old supporters being replaced by new supporters.

We acknowledge that our paper is descriptive and has many limitations. An obvious limitation is the unreliable measure of authoritarianism. Additionally, we measured voting intention and not actual voting; these can sometimes differ, especially given the face-to-face nature of our data collection as compared to the secrecy of actual voting. We acknowledge that we cannot really be certain of what the most powerful underlying causal factors driving increasing anti-immigrant sentiment are. An alternative explanation for increased anti-immigrant sentiment could be the increasing spread of political misinformation and propaganda in online settings; partisan communities of like-minded individuals could be exciting themselves into adopting more and more extreme positions (Pariser, 2011). However, recent results challenge this narrative; at least in Western contexts, exposure to political disagreement on social media is high (Bakshy et al., 2015; Pew Research Center, 2016) and social media does not polarize people’s views (Boxell et al., 2017). Furthermore, if social media echo chambers and political disinformation had, by themselves, increased anti-immigrant sentiment in Hungary, then something similar could have been expected to happen in Western Europe. However, European Social Survey data suggest that overall public attitudes toward refugees (Hatton, 2016) and immigrants (Heath and Richards, 2016) have remained relatively stable in wake of the so-called refugee crisis in 2015. The effects of online propaganda on attitudes, are, naturally, difficult to completely disentangle from the effects of political leadership. This is especially true if the political leadership is responsible for much of the propaganda. Nevertheless, we believe that it is, as in the West (Arceneaux and Johnson, 2015), the behavior of the political elite that changes people’s attitudes, not media communication *per se*.

## Data Availability Statement

The raw data supporting the conclusions of this article will be made available by the authors, without undue reservation.

## Ethics Statement

The studies involving human participants were reviewed and approved by the Faculty of Education and Psychology, Eötvös Loránd University. The patients/participants provided their written informed consent to participate in this study.

## Author Contributions

J-EL contributed to all aspects of work for this article. ZS contributed to conception, data analysis, and the preparation of the manuscript. LK contributed to the data collection and interpretation, and revising the article critically. All authors contributed to the article and approved the submitted version.

## Conflict of Interest

The authors declare that the research was conducted in the absence of any commercial or financial relationships that could be construed as a potential conflict of interest.

## Publisher’s Note

All claims expressed in this article are solely those of the authors and do not necessarily represent those of their affiliated organizations, or those of the publisher, the editors and the reviewers. Any product that may be evaluated in this article, or claim that may be made by its manufacturer, is not guaranteed or endorsed by the publisher.
